# The role of dexamethasone in scorpion venom-induced deregulation of sodium and water transport in rat lungs

**DOI:** 10.1186/s40635-015-0063-0

**Published:** 2015-09-21

**Authors:** Ceila Maria Sant Ana Malaque, Ana Carolina de Bragança, Talita Rojas Sanches, Rildo Aparecido Volpini, Maria Heloisa Shimizu, Meire Ioshie Hiyane, Niels Olsen Saraiva Câmara, Antonio Carlos Seguro, Lucia Andrade

**Affiliations:** Nephrology Department, University of São Paulo School of Medicine, São Paulo, Brazil; Vital Brazil Hospital, Butantan Institute, São Paulo, Brazil; Immunology Department, Biomedical Sciences Institute IV, University of São Paulo, São Paulo, Brazil

**Keywords:** Scorpion venoms, Pulmonary edema/metabolism, Pulmonary alveoli, Cytokines, Toll-like receptor 4

## Abstract

**Background:**

Severe scorpion envenomation can evolve to lung injury and, in some cases, death. The lung injury could be attributed to acute left ventricular failure and increased pulmonary vascular permeability secondary to the release of inflammatory mediators. In clinical practice, corticosteroids have been administered to reduce the early side effects of the anti-venom. We propose to study the effects of *Tityus serrulatus* venom and dexamethasone on pulmonary expression of sodium and water transporters, as well as on the inflammatory response.

**Methods:**

Wistar rats were injected intraperitoneally with saline (control group), dexamethasone, and saline (2.0 mg/kg body weight—60 min before saline injection; dexamethasone + saline group), venom (*T. serrulatus* venom—3.8 mg/kg body weight), or dexamethasone and venom (2.0 mg/kg body weight—60 min before venom injection; dexamethasone + venom group). At 60 min after venom/saline injection, experiments were performed in ventilated and non-ventilated animals. We analyzed sodium transporters, water transporters, and Toll-like receptor 4 (TLR4) by Western blotting, macrophage infiltration by immunohistochemistry, and serum interleukin (IL) by cytokine assay.

**Results:**

In the lung tissue of non-ventilated envenomed animals, protein expression of the epithelial sodium channel alpha subunit (α-ENaC) and aquaporin 5 (AQP5) were markedly downregulated whereas that of the Na-K-2Cl cotransporter (NKCC1) and TLR4 was elevated although expression of the Na,K-ATPase alpha 1 subunit was unaffected. Dexamethasone protected protein expression of α-ENaC, NKCC1, and TLR4 but not that of AQP5. We found that IL-6, IL-10, and tumor necrosis factor alpha were elevated in the venom and dexamethasone + venom groups although CD68 expression in lung tissue was elevated only in the venom group. Among the ventilated animals, both envenomed groups presented hypotension at 50 min after injection, and the arterial oxygen tension/fraction of inspired oxygen ratio was lower at 60 min than at baseline.

**Conclusions:**

Our results suggest that *T. serrulatu*s venom and dexamethasone both regulate sodium transport in the lung and that *T serrulatus* venom regulates sodium transport via the TLR4 pathway.

## Background

In tropical and subtropical countries, scorpion envenomation is common and sometimes fatal, especially among children [1, 2]. The incidence and severity of such envenomation are remarkable in Africa, the Near- and Middle-East, Mexico; Brazil, the Amazon basin, and southern India [1]. In Brazil, approximately 78,000 scorpion stings are reported annually [3]. *Tityus serrulatus* is considered the most medically important scorpion species in Brazil. Although most cases of scorpion envenomation occur in adults, the most severe cases are in children, in whom mortality is also higher [4].

The clinical manifestations of envenomation by scorpions of the genera *Androctonus*, *Leiurus*, *Buthus*, *Centruroides*, and *Tityus* are quite similar, including pain, persistent nausea, vomiting, hypertension, tachycardia, tachypnea, and prostration. Patients presenting with severe envenomation can also progress to heart failure, pulmonary edema, and shock [4–8]. Most of the symptoms and signs of scorpion envenomation have been attributed to the effects of the venom interacting with sodium channels and of neurotransmitters released from autonomic nerve endings [9, 10]. In severe cases, lung injury is common and is frequently the cause of death [11]. Two distinct mechanisms have been suggested to explain the development of pulmonary edema: acute left ventricular failure resulting from massive catecholamine release [12, 13] and increased pulmonary vascular permeability following the release of inflammatory mediators, such as platelet-activating factor, leukotrienes, and prostaglandins [14–16]. Increased serum levels of pro- and anti-inflammatory cytokines, such as interleukin (IL)-1, IL-6, tumor necrosis factor alpha (TNF-α), and IL-10, have also been observed following *T. serrulatus* envenomation in animals and humans [17–20].

Regardless of its pathogenesis, pulmonary edema is resolved by active sodium transport across the alveolar epithelium via apical amiloride-sensitive sodium channels and basolateral Na,K-ATPase. This active vectorial sodium transport produces a transepithelial osmotic gradient that results in passive movement of water from the air spaces into the alveolar interstitium [21, 22]. In some models of acute lung injury, as well as in patients with acute respiratory distress syndrome, the ability of the lungs to clear edema is impaired [23]. In rats injected with the *T. serrulatus* venom, alveolar fluid clearance is decreased by up to 60 %. In addition, the expression and activity of Na,K-ATPase subunits have been shown to decrease in the basolateral membranes of alveolar type II epithelial cells incubated with scorpion venom [24].

Because they can recognize pathogen-associated molecules, Toll-like receptors (TLRs) are key components in human innate immune responses. In contrast with the adaptive immune system, the innate immune system uses TLRs to react rapidly to a wide range of pathogen-associated molecular patterns, without prior exposure. TLRs were initially characterized by their interactions with bacterial ligands and involvement in the cellular activation associated with infection and sepsis. However, recent studies have shown that TLR2 and TLR4 can recognize non-microbial ligands. Once activated, TLRs induce the production of inflammatory cytokines, such as TNF-α, IL-1β, and IL-6, through an intracellular signaling cascade [25].

In Brazil, it is common to administer corticosteroids prior to the administration of anti-venom [4]. Therefore, the objective of the present study was to evaluate, in rats inoculated with *T. serrulatus* venom, the effects that dexamethasone has on the early clinical, biochemical, and ventilatory parameters, on initial molecular changes in the expression of sodium and water transporters, and on the early inflammatory response.

## Methods

### Animals, experimental materials, and procedures

Adult male Wistar rats weighing 215–250 g were obtained from the animal facilities of the University of São Paulo School of Medicine, provided food and water ad libitum, and maintained on a 12/12-h light/dark cycle.

Venom (from *T. serrulatus*), provided by the Butantan Institute (São Paulo, Brazil), was diluted in sterile saline, aliquoted, and stored at −70 °C. Sodium thiopental and dexamethasone were obtained from Cristália (São Paulo, Brazil). We generated a dose–response curve. We found that intraperitoneal (ip) injection of 3.8 mg/kg body weight (BW) of crude scorpion venom-induced rats to severe envenomation. Rats were divided into the following groups: venom, comprising rats receiving 3.8 mg of venom/kg BW (ip); dexamethasone + venom, comprising rats receiving 2.0 mg/kg BW (ip) of dexamethasone [26], 60 min before receiving venom (as above); and control, comprising rats receiving 0.5 ml of saline (ip). Two sets of experiments were performed. In the first set, we used non-ventilated animals (8 control group rats, 13 venom group rats, and 11 dexamethasone + venom group rats). In the second set, we used ventilated animals (7 control group rats, 6 venom group rats, and 5 dexamethasone + venom group rats).

An additional group of control rats receiving only dexamethasone (2.0 mg/kg) was evaluated in order to determine the effects of the drug on biochemical and ventilatory parameters.

All procedures were performed in compliance with Brazilian Ethics Committee for Animal Experimentation (Federal Law n.11.794, October 8th, 2008, Arouca Act), and all experimental procedures were approved by the University of São Paulo School of Medicine Animal Research Committee (number 515/09).

### Non-ventilated animals

#### Procedures

After injection(s), non-ventilated rats were monitored for 60 min, during which time we analyzed clinical parameters such as lacrimation, salivation, dyspnea, and cyanosis. The survivors were anesthetized with sodium thiopental (50 mg/kg BW). The aorta was cannulated with a PE-60 catheter, and blood samples were collected for biochemical analysis and determination of cytokine levels. The lungs were flushed with phosphate-buffered saline (injected into the aorta), excised, and stored at −70 °C for subsequent Western blotting.

#### Biochemical analysis

At 60 min after venom administration, plasma levels of Na, K, urea, creatinine, creatine kinase, lactate dehydrogenase, amylase, and troponin were measured kinetically.

#### Antibodies

The peptide-derived polyclonal antibodies specific to the Na-K-2Cl cotransporter (NKCC1) were kindly supplied by Dr. R. James Turner (National Institute of Dental and Craniofacial Research, Bethesda, MD). The peptide-derived polyclonal antibodies specific to the epithelial sodium channel alpha subunit (α-ENaC), the Na,K-ATPase alpha 1 subunit (α_1_-Na,K-ATPase), aquaporin 5 (AQP5), TLR4, and actin were obtained from Santa Cruz Biotechnology (Santa Cruz, CA), and the peptide-derived monoclonal antibodies specific to CD68 were obtained from ABD Serotec (Raleigh, NC).

#### Membrane fractions

Samples of lungs were homogenized in ice-cold isolation solution containing protease inhibitors. The homogenates were centrifuged, the supernatants were spun, and the resulting pellets, containing membrane fractions enriched with plasma membranes and intracellular vesicles, were suspended in the isolation solution.

#### Western blotting

Samples of membrane fractions were run on 12.5 % polyacrylamide minigels (for AQP5), 10 % polyacrylamide minigels (for α-ENaC, α1-Na,K-ATPase, and TLR4), or 8 % polyacrylamide minigels (for NKCC1). After transfer by electroelution to nitrocellulose membranes (PolyScreen, PVDF Transfer; Life Science Products, Boston, MA, USA), blots were blocked with 5 % milk and 0.1 % Tween 20 in phosphate-buffered saline for 1 h, then incubated with anti-AQP5 antibody (1:500), NKCC1 antibody (1:1000), α_1_-Na,K-ATPase antibody (1:500), α-ENaC antibody (1:100), or TLR4 antibody (1:100). The labeling was visualized with horseradish peroxidase-conjugated secondary antibody (anti-rabbit IgG, diluted 1:5000, or anti-goat IgG, diluted 1:10,000; Sigma) using the enhanced chemiluminescence detection system (Amersham Pharmacia Biotech, Piscataway, NJ, USA). Bands corresponding to protein expression of AQP5, α-ENaC, α1-Na,K-ATPase, NKCC1, TLR4, and actin were quantified by densitometric analysis using Image J software (Research Services Branch, National Institutes of Health, Bethesda, MD, USA). Bands were normalized to actin and are expressed as percentages of the control values.

#### Immunohistochemistry

Fragments of the lungs from rats of all groups were immersed in 4 % paraformaldehyde, fixed for 2 h, post-fixed in Bouin’s solution for 4 h, drained, dehydrated in 70 % ethanol, and processed for paraffin embedding. The samples were sliced into 4-μm histological sections. The sections were incubated overnight at 4 °C with antibodies against CD68 (1:1000) or AQP5 (1:50). The reaction products were detected with an avidin-biotin-peroxidase complex (Vector Laboratories, Burlingame, CA, USA). The color reactions were developed with 3,3-diaminobenzidine (Sigma) and nickel chloride (8 %) in the presence of hydrogen peroxide, and the material was then counterstained with methyl green, dehydrated, and mounted. Infiltrating macrophage/monocyte-positive cells were counted in 30 grid fields (0.087 mm^2^ each). The volume proportion of AQP5 in the alveolar tissue of lung sections was determined by dividing the number of points hitting AQP5 by the total number of points hitting alveolar septa (in 20 fields). Results are expressed as a percentage of positive area per total area of tissue. For counting positive cells, we used the AxioVision program, version 4.8 (Carl Zeiss, Eching, Germany), and we used ImagePro Plus 4.1 (Media Cybernetics, Silver Spring, MD), for measuring area and percentage.

#### Inflammatory cytokines

To determine plasma levels of IL-6, IL-10, and TNF-a, we used a Bio-Plex rat cytokine assay kit (R&D Systems, Minneapolis, MN, USA). The assay was read on the Bio-Plex suspension array system (Bio-Rad, Hercules, CA, USA), and the data were analyzed with Bio-Plex Manager software, version 6.0 (Millipore, Billerica, MA, USA).

### Ventilated animals

#### Procedures

**Fig. 1:**
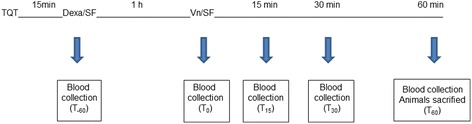
Diagram of the study protocol (ventilated animals)

In ventilated rats, we evaluated the following parameters: the arterial oxygen tension/fraction of inspired oxygen (PaO_2_/FIO_2_) ratio, bicarbonate and plasma glucose (Fig. 1), mean arterial pressure (MAP), and heart rate (HR). We subtracted the PaO_2_/FiO_2_ at 30 and 60 min from the PaO_2_/FiO_2_ before venom injection because that difference (ΔPaO_2_/FiO_2_) is more important than is the absolute PaO_2_/FIO_2_ at either time point.

All animals were submitted of mechanical ventilation at a tidal volume of 10 ml/kg, positive end-expiratory pressure (PEEP) of 3 cmH_2_O, FIO_2_ of 50 %, and respiratory rate of 60 breaths/min. Animals were anesthetized with thiopental (50 mg/kg BW ip) and underwent tracheotomy. The right carotid artery was cannulated with a PE-60 catheter in order to determine MAP, as well as to allow blood sampling at the various time points. The left jugular vein was also cannulated with a PE-60 catheter for the infusion of drugs and venom. The rats were connected to a small animal ventilator (Atlanta; Takaoka, São Paulo, Brazil). Pancuronium (1 ml/kg BW ip) was then administered. To allow the animals to stabilize, we did not initiate the experiment until 15 min after the tracheotomy (Fig. 1). After stabilization, the control and venom groups received saline, whereas the dexamethasone + venom group received dexamethasone diluted in saline. In order to evaluate the effects of dexamethasone in the ventilated animals, we determined serum glycemia and arterial blood gases at 60 min after the initiation of mechanical ventilation, immediately prior to envenomation.

#### Arterial blood sampling and hemodynamic evaluation

Arterial blood gases and glucose were analyzed with a blood gas analyzer (ABL800 FLEX; Radiometer, Copenhagen, Denmark). In addition, heart rate and MAP were continually monitored with an invasive constant monitoring probe (MP100; Biopac Systems, Goleta, CA, USA).

### Statistical analysis

All quantitative data are expressed as mean ± SEM. Comparisons between proportions were analyzed by chi-square test or Fisher’s exact test. Differences among the means of multiple parameters were analyzed by ANOVA followed by the Student-Newman-Keuls test. Values of *p* ≤ 0.05 were considered statistically significant.

## Results

### Non-ventilated animals

#### Clinical data

Some venom-injected rats exhibited systemic manifestations, as early as 30 min after the injection, including lacrimation, salivation, dyspnea, cyanosis, and chromodacryorrhea. Of the 13 venom group rats, 1 (7.7 %) died before the end of the 60-min observation period and 4 (30.8 %) showed no clinical manifestations consistent with severe poisoning, compared with 2 (18.2 %) and 4 (36.4 %) of the 11 dexamethasone + venom group rats (NS).

#### Biochemical data

Serum potassium levels were lower in the venom and dexamethasone + venom groups than in the control and dexamethasone groups. Serum amylase levels were higher in the dexamethasone + venom group than in the other groups (Table 1).

**Table 1:** Biochemical data for non-ventilated rats after administration of saline, dexamethasone, *T. serrulatus* venom, or dexamethasone plus *T. serrulatus* venom

Group	Na	K	Urea	Creatinine	Amylase	CK	LDH	Troponin
	mEq/L	mEq/L	mg/dl	mg/dl	U/L	U/L	U/L	ng/ml
Control	143.1 ± 2.3	4.3 ± 0.2	38.9 ± 2.6	0.3 ± 0.0	2210.0 ± 89.5	572.4 ± 66.1	623.0 ± 187.2	0.2 ± 0.2
Dx	148.5 ± 0.96	3.7 ± 0.2	46.8 ± 3.8	0.2 ± 0.0	2268.0 ± 146.3	532.0 ± 84.7	385.0 ± 49.6	0.0 ± 0.0
Vn	140.6 ± 2.4	3.2 ± 0.1*	44.4 ± 5.4	0.3 ± 0.0	2311.0 ± 112.2	630.8 ± 118.5	525.4 ± 129.2	0.1 ± 0.1
Dx + Vn	142.3 ± 1.4	3.4 ± 0.2*	55.5 ± 13.1	0.3 ± 0.1	3213.0 ± 338.5^**^	761.0 ± 140.8	586.3 ± 97.4	0.0 ± 0.0

*CK* creatine kinase, *LDH* lactate dehydrogenase, *Dx* dexamethasone, *Vn* venom, Dx + Vn dexamethasone + venom

*p < 0.05 compared with control

^**^ *p* < 0.05 compared with the other groups

#### Sodium and water transporters in lungs

**Fig. 2:**
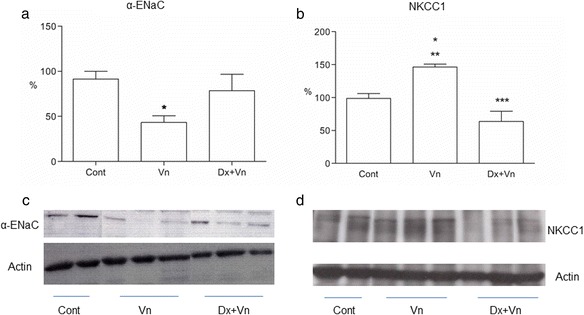
Alpha-ENaC and NKCC1 lung expression. Semiquantitative immunoblotting of membrane fractions prepared from lung tissue samples from rats in the groups venom (Vn, *n* = 8), dexamethasone + venom (Dx + Vn, *n* = 6), and control (Cont, *n* = 4). **a** Densitometric analysis revealing significantly lower α-ENaC expression in the Vn group and no statistical difference between the Dx + Vn and Cont groups. *P < 0.05 compared with Cont and Dx + Vn. **b** Immunoblots reacted with anti-α-ENaC, revealing a 90-kDa band. **c** Densitometric analysis showing that NKCC1 expression was significantly higher in the Vn group and significantly lower in the Dx + Vn group. *P < 0.01 vs. Cont; **P < 0.001 vs. Dx + Vn; ***P < 0.05 vs. Cont. **d** Immunoblots reacted with anti-NKCC1, revealing a 170-kDa band and anti-Actin, 43-kDa

Figure 2 shows that α-ENaC expression in the venom group (43.2 ± 7.3 %) was lower than in the control group (91.5 ± 8.5 %) and dexamethasone + venom group (78.6 ± 18 %), the differences being significant (*p* < 0.05 for both). Semiquantitative immunoblotting revealed that pulmonary expression of α_1_-Na,K-ATPase was comparable across the venom, dexamethasone + venom, and control groups (102.5 ± 7.2, 102.8 ± 7.6, and 95.3 ± 6.0 %, respectively).

As shown in Fig. 2, pulmonary NKCC1 expression in the venom group (146.2 ± 4.5 %) was significantly higher than in the control group (98.5 ± 7.4 %, *p* < 0.01) and dexamethasone + venom group (63.6 ± 15.6 %, *p* < 0.001), the dexamethasone + venom value being significantly lower than that obtained for the control group (*p* < 0.05).

**Fig. 3:**
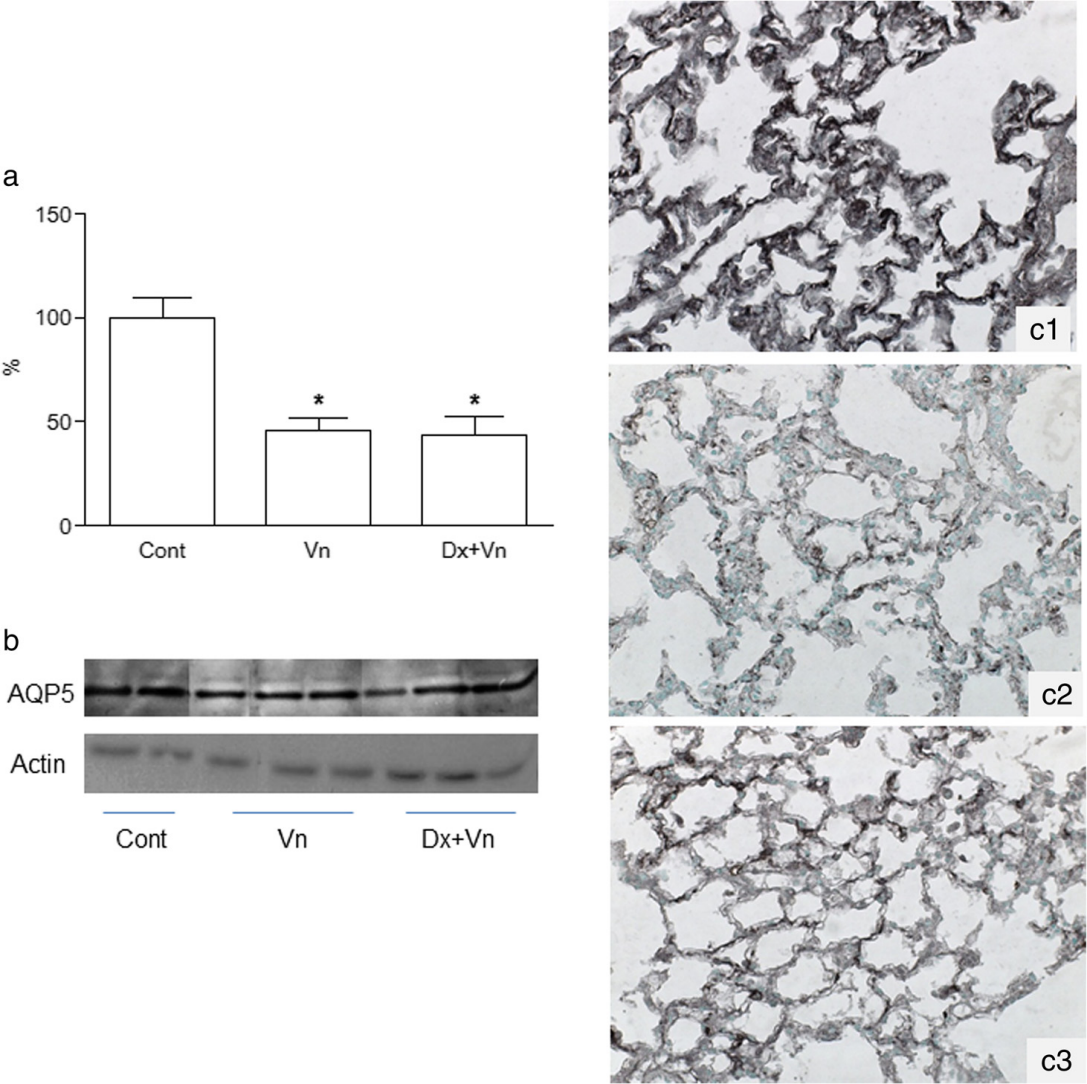
AQP5 lung expression. Semiquantitative immunoblotting of membrane fractions prepared from lung tissue samples from rats in the groups venom (Vn, *n* = 8), dexamethasone + venom (Dx + Vn, *n* = 8), and control (Cont, *n* = 6). **a** Densitometric analysis revealing significantly lower AQP5 expression in the Vn and Dx + Vn groups. *P < 0.05 compared with Cont. **b** Immunoblots reacted with anti-AQP5, revealing a 35-kDa band. *C1-C3* Immunohistochemical localization of AQP5 in lung parenchyma of rats in the groups Cont (*C1*), Vn (*C2*), and Dx + Vn (*C3*) at 60 min after envenomation (magnification, ×400)

Figures 3a, b shows that pulmonary expression of AQP5 was significantly lower in the venom and dexamethasone + venom rats than in the controls (46.0 ± 5.8 and 43.9 ± 8.6 % vs. 100.1 ± 9.2 %, *p* < 0.05). In accordance with the protein expression results, the immunohistochemical staining for AQP5 was less intense and less extensive in the venom and dexamethasone + venom groups than in the control group (Fig. 3C1-C3).

#### Macrophage infiltration

**Fig. 4:**
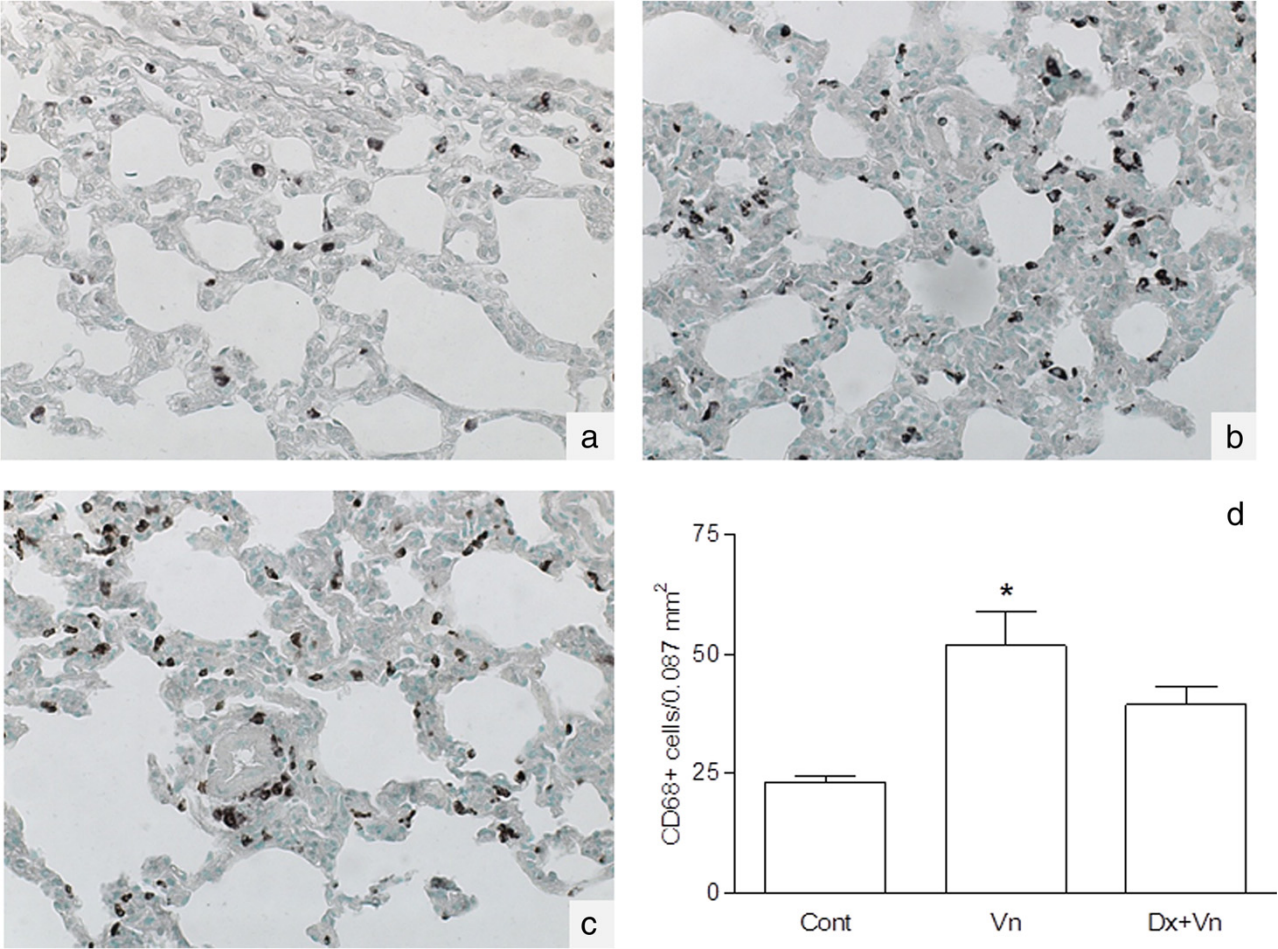
Alveolar macrophage infiltration. Alveolar infiltration by CD68-positive cells (cells/0.087 mm^2^) at 60 min after envenomation in tissue samples from rats in the groups venom (Vn, *n* = 5), dexamethasone + venom (Dx + Vn, *n* = 5), and control (Cont, *n* = 3). Immunostaining for CD68 in the Cont (**a**), Vn (**b**), and Dx + Vn (**c**) groups (magnification, ×400 for all). CD68-positive cell counts (mean ± SEM), showing that alveolar macrophage infiltration was significantly greater in the Vn group (**d**). *P < 0.05 compared with Cont

At 60 min after venom administration, the number of cells presenting CD68 staining for macrophages/monocytes in the lung (Fig. 4) was significantly higher in the venom group than in the control group (51.9 ± 6.9 vs. 23.2 ± 1.3 cells/0.087 mm^2^, *p* < 0.05). The number obtained for the dexamethasone + venom group (39.4 ± 3.8 cells/0.087 mm^2^) did not differ significantly from those obtained for the other groups.

#### TLR4 expression

**Fig. 5:**
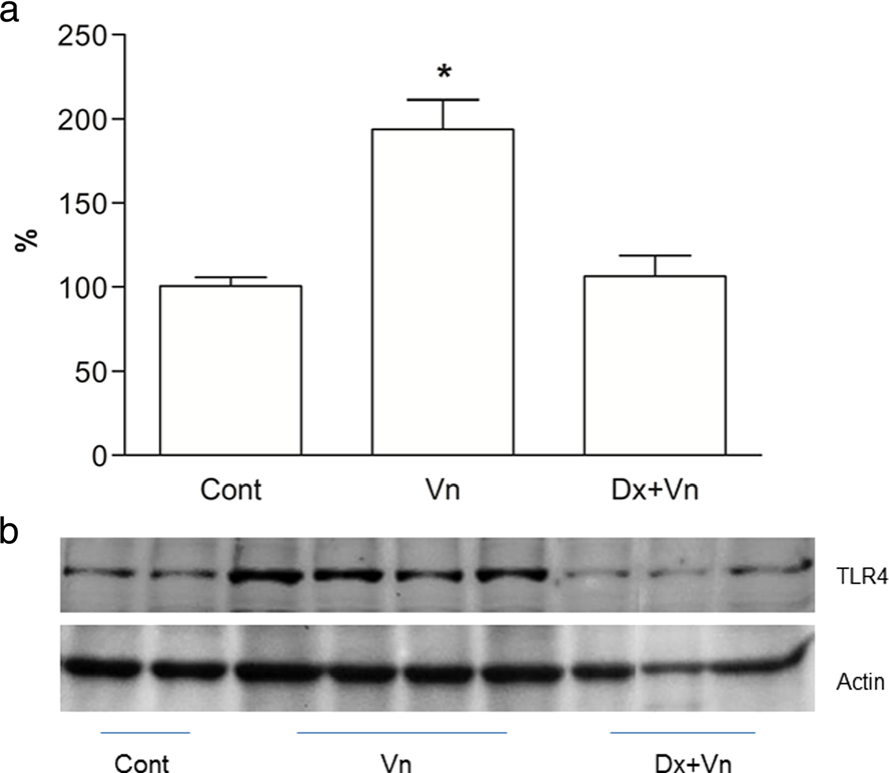
TLR4 lung expression. Semiquantitative immunoblotting of membrane fractions prepared from lung tissue samples from rats in the groups venom (Vn, *n* = 5), dexamethasone + venom (Dx + Vn, *n* = 4), and control (Cont, *n* = 5). **a** Densitometric analysis revealing significantly higher TLR4 expression in the Vn group. *P < 0.05 compared with Cont and Dx + Vn. **b** Immunoblots reacted with anti-TLR4, revealing an 89-kDa band

As shown in Fig. 5, TLR4 expression was significantly higher in venom rats than that in control and dexamethasone + venom rats (194 ± 17.5 % vs. 100.6 ± 5.2 and 106.3 ± 12.3 %, *p* < 0.05 for both).

#### Plasma cytokine levels

Plasma cytokine levels (in pg/ml) were significantly higher in the venom and dexamethasone + venom groups than in the control group—IL-6, 114.9 ± 21.4 and 108.9 ± 8.9 vs. 88.8 ± 49.2; TNF-α, 11.84 ± 0.0 and 11.54 ± 0.0 vs. 5.9 ± 3.3; and IL-10, 42.6 ± 9.0 and 35.7 ± 6.8 vs. 10.0 ± 2.4—(*p* < 0.05 for all).

### Ventilated animals

#### Clinical data

After 60 min on mechanical ventilation (immediately before venom or saline administration) (Fig. 1), the biochemical, respiratory and hemodynamic parameters in the dexamethasone group did not differ from those observed in the control group: glycemia—120.0 ± 4.4 vs. 125.2 ± 4.3 (NS); bicarbonate—18.2 ± 0.7 vs. 18.2 ± 0.4 (NS); ΔPaO_2_/FIO_2_ (T_0_-T_−60_)—7.2 ± 9.7 vs. 10.0 ± 6.0 (NS); MAP—142.0 ± 1.9 vs. 142.0 ± 5.8 (NS); HR—493.8 ± 21.5 vs. 503.0 ± 8.3 (NS).

#### Biochemical data

Hyperglycemia was seen in the venom and dexamethasone + venom groups. In both groups, the levels of glycemia were highest at 30 min after venom injection (Table 2).

**Table 2:** Metabolic parameters in ventilated animals

Group	ΔPaO_2_/FIO_2_		Bicarbonate (mEq/L)			Glucose (mg/dl)		
	T_30_–T_0_	T_60_–T_0_	T_0_	T_30_	T_60_	T_0_	T_30_	T_60_
Control	3.5 ± 3.2	14.0 ± 6.3	21.3 ± 0.6	17.8 ± 0.6	18.5 ± 0.4	119.2 ± 0.9	110.3 ± 8.6	106.7 ± 5.9
Vn	−11.0 ± 6.1	−14.8 ± 4.3*	20.0 ± 0.7	12.8 ± 0.4*	11.5 ± 0.4*	121.8 ± 2.0	249.8 ± 12.3*	189.8 ± 23.0*
Dx + Vn	−14.7 ± 11.9	−9.0 ± 7.9*	19.9 ± 0.4	13.3 ± 1.2*	12.8 ± 1.6*	119.3 ± 4.8	245.3 ± 24.0*	166.8 ± 27.9*

*ΔPaO*_*2*_ */FIO*_*2*_ delta arterial oxygen tension/fraction of inspired oxygen (T_30_ minus T_0_; T_60_ minus T_0_), *T*_*0*_ immediately before envenomation, *T*_*30*_ 30 min after envenomation, *T*_*60*_ 60 min after envenomation, *Vn* venom, *Dx + Vn* dexamethasone + venom

*p < 0.05 compared with control

#### Respiratory and hemodynamic parameters

**Fig. 6:**
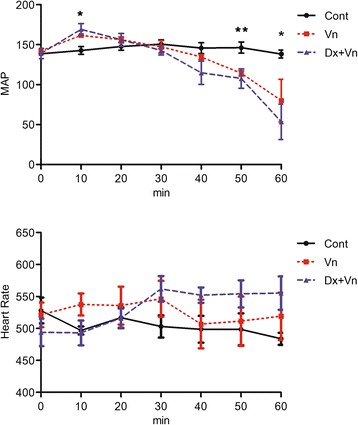
Hemodynamic variation during envenomation. Effect of *Tityus serrulatus* venom administration on mean arterial pressure and heart rate in the venom (Vn), dexamethasone + venom (Dx + Vn) and control (Cont) groups. *P < 0.05 for Dx + Vn vs. Cont, **P < 0.05 for Vn and Dx + Vn vs. Cont)

At 60 min after venom injection, the greater ΔPaO2/FIO2 (i.e., the difference between the PaO_2_/FIO_2_ values obtained immediately before and 60 min after envenomation) indicates that PaO2/FIO2 was worse in both envenomed groups than in the control group (Table 2). At 50 min after venom administration, the animals in both envenomed groups presented hypotension, whereas the control group animals did not. Although we expected the hypotension seen in the envenomed groups to increase heart rates, we found no statistical differences in heart rate among the groups (Fig. 6). However, the mean heart rate was slightly higher in the dexamethasone + venom group, a difference that trended toward significance in comparison with the other groups. In order to analyze the acid–base status, we measured plasma bicarbonate levels. At 30 and 60 min after venom administration, plasma bicarbonate levels were lower in the venom and dexamethasone + venom groups than in the control group (Table 2).

## Discussion

The animal model used in the present study, which involved a high dose of scorpion venom, mimicked the physiological manifestations of severe human envenomation [6, 27], provoking altered expression of sodium and water transporters in lung tissue.

The hyperamylasemia observed in the dexamethasone + venom group might be attributable to synergism between the dexamethasone and the high dose of venom, given that no hyperamylasemia was observed in the venom or dexamethasone groups. This suggests a potential adverse effect of corticosteroid administration in the absence of specific anti-venom administration.

In extremely severe cases of scorpion envenomation, pulmonary edema is common and may lead to death. In some cases, pulmonary edema can persist even 24 h after anti-venom administration [28]. Pulmonary edema is more affected by active sodium transport out of the alveoli than by the reversal of Starling forces. In alveolar epithelial cells, *T. serrulatus* venom downregulates Na,K-ATPase [24]. In our study, *T. serrulatus* venom decreased expression of α-ENaC and AQP5, as well as upregulating basolateral NKCC1, although α_1_-Na,K-ATPase expression was unaffected. Impaired fluid handling can hinder pulmonary function and increase the susceptibility of the lung to injury [22]. Active transport by the Na,K-ATPase pump generates an osmotic driving force favorable to the entrance of sodium via α-ENaC. There is therefore continuous transport of sodium from the lumen into the interstitial space [29]. Volume is regulated primarily by electroneutral cotransporters such as NKCC1, which is found in virtually all cells and mediates the coupled influx of sodium, potassium, and chlorine. The mechanism by which cell shrinkage activates NKCC1 is unknown [30]. We hypothesized that *T. serrulatus* venom might impair pulmonary fluid transport because it decreases α-ENaC and AQP5 expression, as well as increases NKCC1 expression. Although it has been demonstrated that AQP5 knockout mice show the same pulmonary fluid transport as do control mice [31–33], it is possible that, in association with altered sodium transporter expression, decreased AQP5 expression impairs pulmonary fluid transport in severe scorpion envenomation.

In Brazil, it is common to give corticosteroids prior to the administration of anti-venom [4]. A clinical study of pediatric patients with severe scorpion envenomation, comparing those that did and did not receive a corticosteroid together with anti-venom, showed that there were no differences between the groups in terms of mortality or length of stay in the intensive care unit [34]. However, the corticosteroid group patients presented systemic manifestations (indicative of more severe envenomation) at admission, which calls into question the conclusion drawn by the authors—that corticosteroid administration does not improve the evolution of cases of severe scorpion envenomation [34]. Therefore, we tested whether dexamethasone has any regulatory effect on the expression of proteins in experimental envenomation. It has been demonstrated that, in alveolar cells, there is an increase of the α-, β-, and γ-ENaC, as well as Na,K-ATPase, after exposure to dexamethasone [35–38]. In the present study, dexamethasone administration prevented a venom-induced decrease in α-ENaC and a venom-induced increase NKCC1 expression. However, we observed no difference between the venom group and the dexamethasone + venom group in terms of α_1_-Na,K-ATPase expression.

Various studies have shown that, in human and experimental scorpion envenomation, there is inflammatory activation [17–20, 39]. Some authors suggest that, in the presence of systemic inflammation, cytokines mediate sodium transporter expression [40]. We investigated the effects of *T. serrulatus* venom on macrophage infiltration, TLR4 expression in the lung, and serum cytokines. TLRs are expressed in immune cells, such as polymorphonuclear granulocytes, macrophages, dendritic cells, and certain epithelial cells. Engagement of TLR4 initiates signaling through intracellular pathways that lead to activation of transcription factors, including nuclear factor κ-B and interferon regulatory factor 3, that transcribe genes such as pro-inflammatory cytokines and other immunoregulatory molecules [25]. *T. serrulatus* venom induces production of inflammatory mediators in peritoneal macrophages by interacting with TLR2 and TLR4 [41]. Mice inoculated with *T. serrulatus* venom show increased perivascular infiltration of mononuclear cells in lung tissue as soon as 15 min after injection [42]. In the present study, the number of CD68-positive cells in the lung increased by 60 min after venom injection, and dexamethasone had no effect on cell infiltration. However, dexamethasone prevented the venom-induced increase in TLR4 expression. In addition, rats inoculated with *T. serrulatus* venom showed elevated serum levels of IL-6, TNF-α, and IL-10 by 60 min after envenomation, and dexamethasone did not reduce cytokine concentrations to control group levels.

In our model, animals developed early hypotension, metabolic acidosis, and worsening of the PaO_2_/FIO_2_ ratio. Although we observed a decrease in blood pressure, there was no accompanying increase in heart rate, as would be expected. Other authors found that heart rates decreased or not changed significantly after the administration of scorpion venom [43, 44].

## Conclusions

We have demonstrated that *T. serrulatu*s venom impairs the pulmonary expression of sodium and water transporters, as well as increase in inflammatory infiltration and cytokine levels. Together with cardiovascular dysfunction, these initial events might be responsible for the lung injury seen in scorpion envenomation. Our results suggest that *T. serrulatu*s venom and dexamethasone both regulate sodium transport and that TLR4 is one of the pathways by which *T. serrulatus* venom regulates sodium transport. Further preclinical and clinical studies are warranted in order to elucidate these mechanisms.

## Abbreviations

AQP5: aquaporin 5
HR: heart rate
IL: interleukin
MAP: mean arterial pressure
NKCC1: Na-K-2Cl cotransporter
PaO_2_/FIO_2_: arterial oxygen tension/fraction of inspired oxygen ratio
PEEP: positive end-expiratory pressure
TLR: toll-like receptor
TNF-α: tumor necrosis factor alpha
α_1_-Na,K-ATPase: Na,K-ATPase alpha 1 subunit
α-ENaC: epithelial sodium channel alpha subunit
